# Field Tests of Performance and Their Relationship to Age and Anthropometric Parameters in Adolescent Handball Players

**DOI:** 10.3389/fphys.2019.01124

**Published:** 2019-09-06

**Authors:** Mehrez Hammami, Souhail Hermassi, Nawel Gaamouri, Gaith Aloui, Paul Comfort, Roy J. Shephard, Mohamed Souhaiel Chelly

**Affiliations:** ^1^Research Unit (UR17JS01) Sport Performance, Health and Society, Higher Institute of Sport and Physical Education of Ksar Saïd, University of “La Manouba”, Tunis, Tunisia; ^2^Higher Institute of Sport and Physical Education of Ksar Saïd, University of “La Manouba”, Tunis, Tunisia; ^3^Sport Science Program, College of Arts and Sciences, Qatar University, Doha, Qatar; ^4^Directorate of Sport, Exercise and Physiotherapy, University of Salford, Salford, United Kingdom; ^5^Faculty of Kinesiology and Physical Education, University of Toronto, Toronto, ON, Canada

**Keywords:** sitting height, handgrip force, back extensor force, anthropometric characteristics, ball games

## Abstract

Handball performance is influenced by age, anthropometric characteristics, technical skills, tactical understanding, and physical abilities. The aims of this study were (i) to determine differences in anthropometric characteristics and physical performance between adolescent handball players across age categories, and (ii) to determine which anthropometric and maturity variables have the greatest relative importance in fitness for this sport. Seventy-nine male handball players drawn from a team in the elite Tunisian Handball league [U18 (*n* = 10); U17 (*n* = 12); U16 (*n* = 17); U15 (*n* = 18); and U14 (*n* = 22)] volunteered for the investigation. Assessments included sprint performances; change in direction tests (T-half test and Illinois modified test); jumping tests (squat jump; counter movement jump; countermovement jump with aimed arms; five-jump test); medicine ball throwing; handgrip force; back extensor force and selected anthropometric measurements. The individual’s age category affected all measurements, with U17 and U18 players showing larger body measurements and significantly better absolute results on all physical tests than U14, U15 and U16 contestants. Scores for the majority of physical performance tests were closely inter-correlated. We conclude that U17 and U18 players show significantly better absolute results than the younger players on all physical tests. Multiple linear regressions, using block-wise entry, indicate that age is the strongest predictor of jump and sprint performances. Several anthropometric characteristics, including body mass, standing height and lower limb length were closely correlated with performance test scores, but after allowing for age only body mass added to the prediction of jumping ability.

## Introduction

Handball performance is influenced not only by anthropometric characteristics, but also by technical skills, tactical understanding, and physical abilities that develop with a player’s age (Chelly et al., 2010; Kruger et al., 2014; Schwesig et al., 2016). Contestants must undertake repeated periods of high intensity activity, sprinting, jumping, changing direction rapidly, making physical contacts, and throwing they pass the ball, block opponents, and attempt to establish an optimal position for the throwing player, alternating with rapid recovery during periods of low intensity activity (Michalsik et al., 2013; Wagner et al., 2014, 2017, 2018, 2019; Michalsik et al., 2015; Hermassi et al., 2018a, b). The strength and power of both upper and lower limb muscles are important determinants of sprinting, jumping, throwing (Hermassi et al., 2017s) and changing direction rapidly (Hermassi et al., 2017b). It has thus been suggested that field assessments of handball players should include a broad range of measures of sprinting, jumping, ability to change direction and maximal strength (Matthys et al., 2013; Massuca et al., 2015; Haugen et al., 2016; Ortega-Becerra et al., 2018; Wagner et al., 2019). However, there may be considerable redundancy in typical assessments, since performance test scores are often quite closely correlated both with one another and with anthropometric data.

The only relevant previous study of adolescent players (Ortega-Becerra et al., 2018) focused upon a number of physical characteristics affecting throwing performance in 44 male players ranging from elite professionals to under-16 contestants. The present investigation examined widely used field measures (sprint times, change in direction tests, vertical jumping and upper and lower limbs strength) in adolescent handball players across various age categories, looking at the extent of correlations between individual test measures, and examining their relationships to age category and selected anthropometric characteristics (standing and sitting height, lower limb length and percentage body fat). Multiple linear regression analyses (MLR) examined how far measures of maturity and anthropometric characteristics added to the description of ability provided by age alone. Our initial hypotheses were (i) that anthropometric characteristics and physical performance would develop significantly over the age categories studied, and (ii) that a player’s anthropometric characteristics would add to an age-related prediction of physical performance.

## Materials and Methods

### Participants

The study was reviewed and approved by the Institute’s Committee on Research for the Medical Sciences (Manouba University Ethics Committee), in accordance with current national laws and regulations and the Helsinki Declaration. Informed consent was gained from all participants and their parents or guardians after a verbal and a written explanation of the experimental protocol and its potential risks and benefits. Participants were assured that they could withdraw from the trial without penalty at any time.

Seventy-nine male U18 handball players with at least of 5 years playing experience, drawn from a team belonging to the first Tunisian Handball league volunteered to participate in the investigation; details of training experience, playing positions, handedness and maturity status are summarized in Table 2. All were in good health and had passed a medical examination provided by the team physician before commencing the study. Their maturity status was calculated as a maturity offset (Mirwald et al., 2002):

Maturity Offset = −9.236 + 0.000278 leg length × sitting height −0.001663 age × leg length + 0.007216 age × sitting height + 0.02292 weight × height (years)

Players were instructed to avoid any strenuous exercise on the day before testing, and no additional training was conducted on the 2 test days. The training routine comprised repeated ∼90 min training sessions (8 per week for U18; 6 for U17 and U16; 5 per week for U15 and U14), together with a competitive game played on the weekend. Training consisted mainly of tactical skill development (60% of session time) and strength and conditioning routines (40% of session time).

### Experimental Design

We examined differences in anthropometric characteristics and physical performance of adolescent handball players across age categories, looked at test redundancy in terms of inter-correlations between the various performance measures, and finally examined the influence of age and anthropometric characteristics upon performance using both univariate and multi-variate regression equations.

When testing was undertaken, all players had been training for 5 months, and they were already 4 months into the competitive season (January 2017). Two weeks before definitive measurements, two test familiarization sessions were completed. The definitive protocol included anthropometric measures and assessments of sprint performance over 5-, 10-, 20-, and 30-m distances; change in direction tests [T-half test (T-half) and Illinois modified test (Illinois-MT)]; jumping tests [squat jump (SJ); counter movement jump (CMJ); countermovement jump with aimed arms (CMJA); five-jump test (5JT)]; a medicine ball throw; and determinations of handgrip force (HG) and back extensor strength. All test measurements were made at the same time of day, and under the same experimental conditions. Participants maintained their normal intake of food and fluids, but they abstained from physical exercise for 1 day, drank no caffeine-containing beverages for 4 h, and ate no food for 2 h before testing. A 15 min active warm-up comprising running, jumping, sprinting for short distances (10 and 15 m) and mobility exercises, as well as sport-specific drills with or without the ball) preceded each day’s testing, and verbal encouragement ensured maximal effort throughout.

### Testing Schedule

Definitive tests were performed in a fixed order over 3-days. On the first day, anthropometric measurements were followed by vertical jump tests (SJ; CMJ; and CMJA). The second day was devoted to medicine ball testing, Illinois-MT, Back Extensor Strength measurements and 5JT. On the third day, 30 m sprint performance was evaluated, followed by the handgrip test and the T-half test.

#### Anthropometry

Anthropometric measurements included: standing and sitting heights (Holtain stadiometer, Crosswell, Crymych, Pembrokeshire, United Kingdom, accuracy of 1 mm) and body mass (Tanita BF683W scale, Munich, Germany, accuracy of 0.1 kg). The overall percentage of body fat was estimated from the biceps, triceps, subscapular, and suprailiac skinfolds, using the equations of Durnin and Womersley (1974) for adolescent males aged 16.0–19.9 years:

% Body fat = [4.95/(Density − 4.5)] × 100
Where: Density = 1.1533–0.0643 (Log sum of 4 skinfolds) for participants < 17 years old, and
Density = 1.162–0.063 (Log sum of 4 skinfolds) for participants 17- and 19 years old

#### Vertical Jumping

Jump height was assessed by the same investigator, using an infrared photocell mat connected to a digital computer (Optojump System, Microgate SARL, Bolzano, Italy). The optical acquisition system measured contact and flight times during a jump with a precision of 1/1000 s and calculated the jump height from this data. One minute of rest was allowed between the three trials of each test, the highest jump being used in subsequent analyses. Participants were instructed to land with the legs fully extended and then to flex the limbs on landing, to avoid artificially inflating flight-time. Participants began the SJ at a knee angle of 90 degrees, and avoiding any downward movement, they performed a vertical jump by pushing upward, keeping their legs straight throughout. The CMJ began from an upright position, with participants making a rapid downward movement to a knee angle of approximately 90°, arms akimbo and simultaneously beginning to push-off, after being instructed to jump as fast and high as possible. The hands were freely used during the CMJA.

#### Medicine Ball Throw

Medicine ball throws were performed using 21.5 cm diameter 1 and 3 kg rubber medicine balls (Tigar, Pirot, Serbia). All subjects began with a familiarization session. A brief description of the optimal technique was given, suggesting a release angle to achieve a maximum distance of throw (Gillespie and Keenum, 1987). The medicine ball was lightly covered with chalk powder (magnesium carbonate) to absorb sweat and ensure a firm grip on the ball. The talc also marked the floor where the ball landed, allowing a precise measurement of the throwing distance. The sitting player grasped the medicine ball with both hands, and on the given signal forcefully pushed the ball from the chest. The score was measured from the front of the sitting line to the place where the ball landed.

#### Modified Change in Direction Illinois Test

Modified Illinois test (Illinois-MT) outcomes were recorded using an electronic timing system (Microgate SARL, Bolzano, Italy). Two pairs of tripod-mounted timing sensors were set 1 m above the floor and facing each other 3 m apart on either side of the starting and finishing lines. The front foot was positioned on a line 0.20 m in front of the photocell beam. The change in direction area for the Illinois-MT was set-up with four cones. On command, the player sprinted 5 m from a standing position, turns and came back to the starting line; then swerved in and out of the four markers, completing two 5 m sprints to finish the course (Hachana et al., 2014). Participants were told to complete the test as quickly as possible, but no advice is given on technique. They were also instructed not to cut over the markers, but to run around them. If they failed to do this, the trial was stopped and re-attempted after a standard recovery period.

#### Back Extensor Strength

Maximal isometric back extensor strength was measured in kilograms, using back and leg dynamometers (Takei, Tokyo, Japan) as previously described (Hannibal et al., 2006). Participants stood on the dynamometer foot stand with their feet one shoulder-width apart and gripped the handle bar positioned across the thighs. The chain-length of the dynamometer was adjusted so that initially the legs were fully extended and the back was flexed at a 30° angle, positioning the bar at the level of the patella. Participants then stood upright without bending their knees and lifted the dynamometer chain, pulling upward as strongly as possible. Three trials were completed, and the highest score was recorded. A 30-s rest interval was allowed between each trial.

#### Five-Jump Test (5JT)

The 5JT began from an upright standing position, with both feet flat on the ground. Participants tried to cover as much distance as possible with five forward jumps, alternating left- and right-leg ground contacts. The distance covered was measured to the nearest 1 cm using a tape measure (Meylan and Malatesta, 2009).

#### 30 m Sprint Performance

Times over distances of 5-, 10-, 20-, and 30 m were recorded using a series of paired photocells (Microgate, Bolzano, Italy). Participants started from a standing position, with the front foot 0.2 m from the first photocell beam. Three trials were separated by 6–8 min of recovery, with the best result for each distance being noted.

#### Handgrip Force

The subject held the hand dynamometer (Takei, Tokyo, Japan) with the arm at right angles and the elbow by the side of the body. The handle of the dynamometer was adjusted so that the base rested on first metacarpal and the handle rested on the middle of the four fingers. The dynamometer was squeezed maximally, and the contraction was maintained for 5 s. No ancillary body movements were allowed. Two trials were made with each hand, with 1 min of rest between trials. The highest readings were used in subsequent analyses.

#### Modified Change in Direction *t*-Test

The *t*-test was used to determine speed with directional changes such as forward sprinting, left and right shuffling, and back-pedaling. Subjects began the test with both feet behind starting line A (Sassi et al., 2009). Participants sprinted forward to cone B and touched the base of it with their right hand. Facing forward and without crossing feet, they then shuffled to the left to cone C and touched its base with the left hand. They next shuffled to the right to cone D, touching its base with the right hand. They then shuffled back to cone B, touching its base. Finally, they ran back as quickly as possible to line A. If they crossed one foot in front of the other, failed to touch the base of a cone, and/or failed to face forward throughout, they had to repeat the test. Two trials were conducted and the shortest time was recorded.

### Statistical Analyses

All statistical analyses were performed using SPSS version 22.0 for Windows (SPSS Inc., Chicago, IL, United States). The reliabilities of all dependent variables were assessed by calculating two-way mixed intra-class correlation coefficients (Vincent, 1995). Descriptive statistics [mean and standard deviation (SD)] were ascertained for all variables. Comparisons between age groups were performed using a series of one-way analyses of variance. If a significant *F* value was observed, Tukey’s *post hoc* procedure was applied to locate pair-wise differences. Pearson’s product moment correlation was calculated and used to determine relationships between all tests.

Multiple linear regressions (MLR) were calculated using a hierarchical block-wise entry method. Firstly, we tested how much variance our measure of maturity contributed to a simple age prediction of each variable. Then we analyzed how much each of a sequence of anthropometric variables supplemented this description, with the order of entry of predictors into the equation selected on the basis of univariate correlations with the performance variable in question and knowledge of past work. The number of physical performance variables was reduced for these analyses. Individual data for a characteristic such as sprinting were arbitrarily weighted, based on their correlations with anthropometric data (Table 5). Performance measures were then expressed as a percentage of the corresponding group mean (performance for individual − mean performance) × 100)/mean performance, as shown in the following examples:

Composite Sprint score = (aS5m% + bS10m% + cS20m% + dS30m%).
Composite change in direction score = (aT-half% + bIllinois-MT%).
Composite jump score = (aSJ% + bCMJ% + cCMJA% + d5JT%).
Composite strength score = (aMedicine Ball% + bHandgrip right% + cHandgrip left% + dBack Extensor Strength%).

Normality of all data sets was checked using the Kolmogorov–Smirnov test. Multicollinearity was estimated by a variance inflation factor (VIF), with a VIF > 10 indicating excessive multicollinearity. Levene’s test checked the homogeneity of variance, and scatter plots tested the linearity assumption.

## Results

### Preliminary Analysis of the Data

Multicollinearity was tested, and height was excluded from the regression models because its VIF was > 10. Levene’s test showed equal variance across samples, and the oval shape of scatter plots test showed linearity of the data. All performance measurements reached an acceptable level of reliability (Table 1; *r* > 0.80). All variables showed a normal distribution.

**TABLE 1:** Intra-class correlation coefficients and coefficients of variation for measures of physical performance.

**Performance test**	**ICC**	**95%CI of ICC**	**CV**
5 m	0.847	0.760–0.902	4.8
10 m	0.983	0.973–0.989	5.2
20 m	0.996	0.993–0.997	6.8
30 m	0.967	0.948–0.979	7.3
T-half	0.987	0.980–0.992	4.3
Illinois-MT	0.952	0.926–0.970	2.7
SJ	0.921	0.876–0.949	15.5
CMJ	0.984	0.975–0.990	14.8
CMJA	0.926	0.884–0.953	13.9
5JT	0.990	0.984–0.993	17.1
Medicine ball throw	0.947	0.917–0.966	20
Handgrip force right	0.975	0.933–0.973	17.1
Hand grip force left	0.902	0.846–0.937	16
Back extensor strength	0.967	0.948–0.979	12.3

*5JT, five-jump test; CI, confidence intervals; CMJ, counter-movement jump; CMJA, counter-movement jump aimed arms; CV, coefficient of variation; ICC, intra-class correlation coefficient; MT, modified test; SJ, squat jump.*

### Age Effects

There were significant main effects of age for all measurements of both physical characteristics (Table 2) and performance test scores (Table 3) and the majority of physical performance measures showed moderate to very large associations (Table 4). Chronological age had a consistently larger univariate effect on all variables than the age at peak height velocity (Table 5). The U17 and U18 age categories showed significantly larger anthropometric dimensions and larger absolute values for all physical test scores than the U14, U15, and U16 groups. The U16, U17 and U18 groups also performed significantly better than the U14 and U15 for all sprint COD times (Table 3). A consistent age trend was also seen in vertical and five-jump tests; although U17 and U18 players did not differ statistically from each other, significant inter-group differences were found for U 14, U 15, and U 16 players (Table 3).

**TABLE 2:** Comparison of physical characteristics across age categories.

**Player category**	**U14 (*n* = 22)**	**U15 (*n* = 18)**	**U16 (*n* = 17)**	**U17 (*n* = 12)**	**U18 (*n* = 10)**
Age (years)	13.8 ± 0.3a^∗∗∗^b^∗∗∗^c^∗∗∗^d^∗∗∗^	14.7 ± 0.3a^∗∗∗^b^∗∗∗^c^∗∗∗^	15.8 ± 0.3a^∗∗∗^b^∗∗∗^	16.6 ± 0.3a^∗∗∗^	17.7 ± 0.3
APHV (years)	14.1 ± 0.4a^∗∗∗^b^∗∗∗^c^∗∗∗^	14.1 ± 0.3a^∗∗∗^b^∗∗∗^c^∗∗∗^	14.7 ± 0.34	15.0 ± 0.5	15.0 ± 0.4
Body mass (kg)	68.2 ± 4.4a^∗∗∗^b^∗^	68.7 ± 3.8a^∗∗∗^	69.9 ± 5.8a^∗∗∗^	74.0 ± 8a^∗∗∗^	86.3 ± 5.9
Height (cm)	167.9 ± 5.9a^∗∗∗^b^∗∗∗^c^∗∗∗^d^∗∗∗^	175.7 ± 5.3a^∗∗^	179.3 ± 2.8	180.1 ± 3.3	182.5 ± 2.4
Sitting height (cm)	79.1 ± 3.4a^∗∗∗^b^∗^c^∗^d^∗^	82.0 ± 1.9a^∗∗^	81.8 ± 2.2a^∗∗^	82.2 ± 3.5a^∗∗^	86.2 ± 2.2
Lower limb length (cm)	88.8 ± 4.6a^∗∗∗^b^∗∗∗^c^∗∗∗^d^∗∗∗^	93.7 ± 4.2b^∗^c^∗^	97.4 ± 1.8	97.9 ± 2.2	96.3 ± 1.4
Body fat%	23.1 ± 7.2	21.6 ± 7.8	19.3 ± 6.2	21 ± 5.3	17.7 ± 7.6
Training experience (years)	5.4 ± 0.5	5.7 ± 0.5	6 ± 0.9	6.5 ± 1	7.8 ± 0.8
Right handed	16	13	11	10	8
Left handed	6	5	6	2	2
Back players	8	6	6	3	3
Wing players	7	5	5	4	3
Pivots players	4	4	3	3	2
Goal-keepers	3	3	3	2	2

*APHV, age at peak height velocity; a, significantly less than U18; b, significantly less than U17; c, significantly less than U16; d, significantly less than U15; n, number of subjects; U, under. ^∗^*p* < 0.05; ^∗∗^*p* < 0.01; ^∗∗∗^*p* < 0.001.*

**TABLE 3:** Comparison of athletic performance of study participants across age categories.

**Age category**	**U14 (*n* = 22)**	**U15 (*n* = 18)**	**U16 (*n* = 17)**	**U17 (*n* = 12)**	**U18 (*n* = 10)**
**Sprint times**					
5 m (s)	1.22 ± 0.05a^∗∗∗^b^∗∗∗^c^∗∗∗^	1.21 ± 0.06a^∗∗^b^∗∗^c^∗∗^	1.15 ± 0.04	1.14 ± 0.02	1.14 ± 0.05
10 m (s)	2.10 ± 0.07a^∗∗∗^b^∗∗∗^c^∗∗∗^	2.08 ± 0.09a^∗∗∗^b^∗∗∗^c^∗∗∗^	1.91 ± 0.05	1.95 ± 0.05	1.91 ± 0.05
20 m (s)	3.68 ± 0.12a^∗∗∗^b^∗∗∗^c^∗∗∗^	3.73 ± 0.19a^∗∗∗^b^∗∗∗^c^∗∗∗^	3.30 ± 0.05	3.32 ± 0.05	3.34 ± 0.21
30 m (s)	5.28 ± 0.19a^∗∗∗^b^∗∗∗^c^∗∗∗^d^∗^	5.03 ± 0.41a^∗∗∗^b^∗∗∗^c^∗∗∗^	4.68 ± 0.12	4.66 ± 0.08	4.58 ± 0.15
**Times for change in direction tests**					
T-half (s)	7.33 ± 0.27a^∗∗∗^c^∗∗^	7.16 ± 0.39a^∗∗^	7.01 ± 0.16	7.10 ± 0.16a^∗^	6.78 ± 0.13
Illinois-MT (s)	13.41 ± 0.25a^∗∗∗^b^∗∗∗^c^∗∗∗^	13.21 ± 0.22a^∗∗∗^b^∗∗∗^c^∗∗^	12.93 ± 0.26a^∗∗^	12.84 ± 0.16	12.60 ± 0.17
**Vertical jump heights**					
SJ (cm)	25.7 ± 1.7a^∗∗∗^b^∗∗∗^	26.1 ± 3.6a^∗∗∗^b^∗∗^	28.2 ± 4.8a^∗∗^	30.7 ± 4.5	33.5 ± 2.0
CMJ (cm)	27.8 ± 2.2a^∗∗∗^b^∗∗^	27.8 ± 3.5a^∗∗∗^b^∗^	30.1 ± 5.1a^∗∗^	32.7 ± 4.2	35.2 ± 2.7
CMJA (cm)	31.3 ± 1.5a^∗∗∗^b^∗∗^	32.1 ± 3.5a^∗∗∗^b^∗^	33.9 ± 5.3a^∗^	36.9 ± 5.9	39.0 ± 2.5
**Horizontal jump**					
5JT (m)	8.1 ± 0.4a^∗∗∗^b^∗∗∗^c^∗∗∗^	8.4 ± 0.7a^∗^b^∗∗∗^c^∗∗∗^	10.5 ± 0.7	10.5 ± 0.6	10.0 ± 3
**Strength**					
Medicine Ball Throw (m)	3.3 ± 0.3a^∗∗∗^b^∗∗∗^c^∗∗∗^d^∗∗^	3.7 ± 0.2a^∗∗∗^b^∗∗∗^c^∗∗∗^	4.9 ± 0.4	5.2 ± 0.3	5.0 ± 0.4
Hand grip force right (N)	356 ± 23a^∗∗∗^b^∗∗∗^c^∗∗^	386 ± 25a^∗∗∗^b^∗∗^	416 ± 68a^∗∗∗^	463 ± 88	504 ± 32
Hand grip force left (N)	339 ± 18a^∗∗∗^b^∗∗∗^c^∗∗^	370 ± 30a^∗∗∗^b^∗∗^	389 ± 62a^∗∗∗^	436 ± 70	467 ± 40
Back extensor force (N)	1154 ± 74a^∗∗^b^∗∗^	1241 ± 84	1174 ± 180a^∗^b^∗^	1342 ± 217	1340 ± 86

*5JT, five jump test; a, significantly different from U18; b, significantly different from U17; c, significantly different from U16; CMJ, counter movement jump; CMJA, counter movement jump aimed arms; d, significantly different from U15; MT, modified test; n, number; SJ, squat jump; U, under. ^∗^*p* < 0.05; ^∗∗^*p* < 0.01; ^∗∗∗^*p* < 0.001.*

**TABLE 4:** Correlation matrix for measures of physical performance.

5 m (s)	**5 m**												
10 m (s)	**0.85 ^∗∗∗^**	**10 m**											
20 m (s)	**0.70 ^∗∗∗^**	**0.83 ^∗∗∗^**	**20 m**										
30 m (s)	**0.63 ^∗∗∗^**	**0.74 ^∗∗∗^**	**0.66 ^∗∗∗^**	**30 m**									
T-half (s)	0.49 ^∗∗^	**0.52 ^∗∗∗^**	0.40 ^∗^	0.51 ^∗∗^	**T-half**								
Illinois-MT (s)	0.67 ^∗∗^	**0.72 ^∗∗∗^**	**0.60 ^∗∗∗^**	**0.56 ^∗∗∗^**	0.48 ^∗∗^	**Illinois-MT**							
SJ (cm)	−0.48 ^∗∗^	**−0.53 ^∗∗∗^**	−0.50 ^∗∗^	−0.42 ^∗^	−0.41 ^∗^	**−0.48 ^∗∗∗^**	**SJ**						
CMJ (cm)	−0.45 ^∗∗^	−0.50 ^∗∗^	−0.50 ^∗∗^	−0.43 ^∗∗^	−0.32	**−0.47 ^∗∗∗^**	**0.91 ^∗∗∗^**	**CMJ**					
CMJA (cm)	−0.46 ^∗∗^	−0.50 ^∗∗^	−0.49 ^∗∗^	−0.40 ^∗^	−0.34	**−0.48 ^∗∗∗^**	**0.86 ^∗∗∗^**	**0.86 ^∗∗∗^**	**CMJA**				
5JT (m)	−0.52 ^∗∗^	**−0.60 ^∗∗∗^**	**−0.54 ^∗∗∗^**	**−0.57 ^∗∗∗^**	−0.37	**−0.50 ^∗∗∗^**	0.42 ^∗∗^	0.43 ^∗∗^	0.29	**5JT**			
Medicine Ball Throw (m)	**−0.55 ^∗∗∗^**	**−0.70 ^∗∗∗^**	**−0.74 ^∗∗∗^**	**−0.74 ^∗∗∗^**	−0.40 ^∗^	**−0.66 ^∗∗∗^**	**0.50 ^∗∗∗^**	**0.48 ^∗∗∗^**	0.46 ^∗∗^	**0.61 ^∗∗∗^**	**Medicine Ball Throw**		
Hand grip test right (N)	**−0.50 ^∗∗∗^**	**−0.54 ^∗∗∗^**	−0.46 ^∗∗^	**−0.54 ^∗∗∗^**	−0.40 ^∗^	**−0.59 ^∗∗∗^**	**0.57 ^∗∗∗^**	**0.50 ^∗∗∗^**	0.47 ^∗∗^	0.43 ^∗∗^	**0.70 ^∗∗∗^**	**Hand grip right**	
Hand grip test left (N)	**−0.48 ^∗∗∗^**	**−0.48 ^∗∗∗^**	−0.40 ^∗^	**−0.51 ^∗∗∗^**	−0.38 ^∗^	**−0.60 ^∗∗∗^**	**0.57 ^∗∗∗^**	**0.52 ^∗∗∗^**	**0.49 ^∗∗∗^**	0.41 ^∗^	**0.67 ^∗∗∗^**	**0.86 ^∗∗∗^**	**Hand grip left**
Back Extensor Strength (N)	−0.31	−0.18	−0.18	−0.28	−0.19	−0.31	0.42 ^∗^	0.42 ^∗^	0.44 ^∗∗^	0.21	0.40 ^∗^	**0.53 ^∗∗∗^**	**0.66 ^∗∗∗^**

*5JT, five jump test; CMJ, counter movement jump; CMJA, counter movement jump aimed arms; MT, modified test; SJ, squat jump; ^∗^*p* < 0.05; ^∗∗^*p* < 0.01; ^∗∗∗^*p* < 0.001. High correlations are highlighted.*

**TABLE 5:** Correlations between age. age at peak height velocity, anthropometric parameters and measures of physical performance.

	**5 m**	**10 m**	**20 m**	**30 m**	**T-half**	**Illinois MT**	**SJ**	**CMJ**	**CMJA**	**5JT**	**Medicine ball throw**	**HG force right hand**	**HG force left hand**	**BEF**
Age	**−0.59 ^∗∗∗^**	**−0.71 ^∗∗∗^**	**−0.69 ^∗∗∗^**	**−0.73 ^∗∗∗^**	**−0.54 ^∗∗∗^**	**−0.78 ^∗∗∗^**	**0.63 ^∗∗∗^**	**0.60 ^∗∗∗^**	**0.59 ^∗∗∗^**	**0.55 ^∗∗∗^**	**0.84 ^∗∗∗^**	**0.72^∗∗∗^**	**0.69 ^∗∗∗^**	0.40^∗∗^
APHV	**−0.43 ^∗∗∗^**	**−0.51 ^∗∗∗^**	**−0.62 ^∗∗∗^**	**−0.51 ^∗∗∗^**	−0.20	**−0.52 ^∗∗∗^**	**0.41 ^∗∗∗^**	**0.44 ^∗∗∗^**	**0.47 ^∗∗∗^**	**0.42 ^∗∗∗^**	**0.65 ^∗∗∗^**	**0.41 ^∗∗∗^**	**0.41 ^∗∗∗^**	0.29 ^∗∗^
Body mass	−0.27	−0.38 ^∗^	−0.31	**−0.45 ^∗∗∗^**	**−0.47 ^∗∗∗^**	**−0.45 ^∗∗∗^**	0.31	0.28	0.21	0.12	**0.44 ^∗∗∗^**	**0.56 ^∗∗∗^**	**0.50 ^∗∗∗^**	0.29
Standing height	**−0.50 ^∗∗∗^**	**−0.60 ^∗∗∗^**	**−0.46 ^∗∗∗^**	**−0.66 ^∗∗∗^**	**−0.61 ^∗∗∗^**	**−0.62 ^∗∗∗^**	**0.44 ^∗∗∗^**	0.36 ^∗^	0.35 ^∗^	**0.51 ^∗∗∗^**	**0.66 ^∗∗∗^**	**0.53 ^∗∗∗^**	**0.51 ^∗∗∗^**	0.26
Sitting height	−0.33^∗^	−0.42 ^∗∗^	−0.23	−0.42 ^∗∗^	**−0.53 ^∗∗∗^**	**−0.49 ^∗∗∗^**	0.38 ^∗∗^	0.31	0.26	0.27	0.42 ^∗∗^	**0.52 ^∗∗∗^**	**0.48 ^∗∗∗^**	0.21
Lower limb length	**−0.48 ^∗∗∗^**	**−0.56 ^∗∗∗^**	**−0.49 ^∗∗∗^**	**−0.56 ^∗∗∗^**	**−0.50 ^∗∗∗^**	**−0.54 ^∗∗∗^**	0.35 ^∗^	0.30	0.32 ^∗^	**0.53 ^∗∗∗^**	**0.64 ^∗∗∗^**	0.39 ^∗∗^	0.39 ^∗∗^	0.22
Body fat%	0.16	0.20	0.18	0.06	−0.01	0.18	−0.30	−0.28	−0.26	−0.26	−0.16	−0.10	−0.12	−0.08

*APHV, age at peak height velocity; 5JT, five jump test; BEF, Back extensor force; CMJ, counter movement jump; CMJA, counter movement jump aimed arms; HG, handgrip; MT, modified *t*-test; SJ, squat jump; ^∗^*p* < 0.05; ^∗∗^*p* < 0.01; ^∗∗∗^*p* < 0.001. High correlations are highlighted.*

### Test Redundancy

The correlation matrix showed that sprint-times over distances of 5–30 m were closely correlated with each other as were the standing jump, counter-movement jump score with and without use of the arms.

### Relationships Between Anthropometric Characteristics and Physical Performance

The majority of physical performance measures showed moderate to very large univariate associations with most anthropometric characteristics (Table 5), correlations being particularly strong for lower limb length, body mass, and standing height. However, back extensor strength did not influence sprinting or COD performance. Further, age, height, and lower limb length were significantly correlated with the results of all physical tests (Table 5). Body mass was also significantly correlated with the majority of physical performance measures except CMJ, CMJA, and 5JT. In contrast, body fat percentage (over the range of body fat values found in these players) was not correlated with any of the physical performance scores (Table 5).

### Multiple Regression Analyses

Some 59.3% of the variance in composite sprint score was attributable to age. After inclusion of this variable, no other potential terms in the prediction equation achieved statistical significance (Table 6). The equation for prediction of sprinting performance was thus:

**TABLE 6:** Multiple regression analyses for composite jump scores.

**Model**	**R**	**R square**	**Adjusted R square**	**SE of the estimate**	**Sig. F change**
Model 1: Age	0.700	0.490	0.483	9.262	<0.001
Model 2: Age + APHV	0.701	0.491	0.477	9.314	0.718
Model 3: Age + body mass	0.739	0.546	0.528	8.854	0.003
Model 4: Age + body mass + LLL	0.739	0.546	0.522	8.913	0.915
Model 5: Age + body mass + body fat	0.743	0.553	0.535	8.790	0.204

*LLL: lower limb length.*

Composite sprint score (%) = −3.04 Age (year) + 46.6

In terms of the composite jump score, 48.3% of the variance was explained by calendar age. Addition of the maturity variable (APHV) did not significantly change the prediction (Table 7). Body mass added a significant 4% to the description of variance, but after introduction of this variable, neither leg length nor body fat content added significantly to the regression. The jump score could thus be predicted using the equation:

**TABLE 7:** Multiple regression analyses for composite sprint scores.

**Model**	**R**	**R square**	**Adjusted R square**	**SE of the estimate**	**Sig. F change**
Model 1: Age	0.770	0.593	0.588	3.451	<0.001
Model 2: Age + APHV	0.773	0.597	0.586	3.459	0.427
Model 3: Age + body mass	0.779	0.607	0.591	3.439	0.170
Model 4: Age + LLL	0.782	0.611	0.601	3.396	0.065
Model 5: Age + body fat	0.771	0.594	0.583	3.471	0.740

*LLL: lower limb length.*

Composite jump score (%) = 8.43 Age (year) − 0.48 Body mass (kg) − 94.6

For the composite change in direction score, age, age at peak height velocity and leg length all contributed to the description of variance (Table 8), with the final equation contributing 59.3% of the variance in performance:

**TABLE 8:** Multiple regression analyses for composite COD scores.

**Model**	**R**	**R square**	**Adjusted R square**	**SE of the estimate**	**Sig. F change**
Model 1: Age	0.732	0.535	0.529	2.094	<0.001
Model 2: Age + APHV	0.761	0.580	0.568	2.005	0.006
Model 3: Age + APHV + LLL	0.780	0.609	0.593	1.946	0.020
Model 4: Age + APHV + LLL + body fat	0.784	0.615	0.594	1.945	0.299

*LLL: lower limb length.*

Composite change in direction score (%) = −1.82 Age (year) + 1.66 APHV (year) – 1.36 Lower limb length (cm) + 16.8

Fort the composite strength scores, 63.8% of the variance was described by age, with none of the other variables contributing to this description (Table 9). Thus, Composite strength score (%) = 8.23 Age (year) − 126.4

**TABLE 9:** Multiple regression analyses for composite strength scores.

**Model**	**R**	**R square**	**Adjusted R square**	**SE of the estimate**	**Sig. F change**
Model 1: Age	0.799	0.639	0.633	8.508	<0.001
Model 2: Age + APHV	0.800	0.640	0.631	8.538	0.495
Model 3: Age + body mass	0.800	0.641	0.626	8.591	0.811
Model 4: Age + LLL	0.800	0.641	0.621	8.648	0.894
Model 5: Age + body fat.	0.801	0.642	0.617	8.893	0.823

*LLL: lower limb length.*

## Discussion

Aspects of the present findings that merit specific comment include the issue of test redundancy, the impacts of age and maturity upon performance in handball, correlations of performance with anthropometric characteristics, the influence of playing position, and finally some strengths and limitations in the research to date.

### Test Redundancy

The close correlation observed between many of the performance measures used in this study highlights a substantial redundancy in the tests that are presently used in assessments of performance for team sports; inter-correlations are particularly close for the four sprint times and for the several measures of jumping performance (Table 4). Others, also, have commented on such inter-relationships and test redundancies (Chaouachi et al., 2009; Chelly et al., 2010; Schwesig et al., 2017; Ortega-Becerra et al., 2018). There is a need to use techniques such as factor analysis to discern underlying structures and measurements or measurement combinations that are aligned with specific components of actual game performance. This would simplify the task both of measuring laboratories and those practitioners who must interpret the resulting data.

### Age Effects

Age is an important variable for handball players (Lidor et al., 2005). Our age comparisons were admittedly cross-sectional in nature, but selection pressures were similar for each age category, and effects from social changes and secular trends to an increase of standing height at any given age are unlikely to have had a major influence over the brief 5-year interval considered here.

There are marked differences in both anthropometric characteristics (Table 2) and physical performance (Table 3) between age categories, and a large part of the total variance in performance variables is described by calendar age (Tables 6–9). This reflects not only the impact of physical growth, but also the accumulation of training, technique and playing experience (Helsen et al., 1998; Salinero et al., 2014). Moreover, in the Tunisian teams, the number and content of training sessions differed between age categories, with 8 (90-min) sessions per week for U18, and 6 training sessions for U17 and U16, but only 5 sessions per week for U15 and U14. Further, the isometric strength training session was reduced for the U 14 category, with loads between 40 to 60% 1-RM, whereas for U18 the strength training involved loads varying from 40 to 120% of 1RM (eccentric contraction). Finally, differences in the percentage of body fat between age categories might have influenced physical performance, since U14 players tended to have a higher percentage of body fat than the other age categories (Table 2).

Inter-individual differences of calendar and biological ages within a given playing category create a relative age effect (Gutierrez Diaz Del Campo, 2010; Prieto-Ayuso et al., 2015), first seen around 12 years of age (Helsen et al., 1998; Gómez-López et al., 2017) and diminishing in the late teens. Those born early after the cut-off date for a given age category have an advantage both in selection and in subsequent performance (Musch and Grondin, 2001; Sherar et al., 2007; Schorer et al., 2009). Consequently, they receive more attention, better training facilities, and more training time (Helsen et al., 2005). In contrast, athletes who are born in the last months of a given age category are often not selected for teams and tend to abandon their sport (Barnsley and Thompson, 1988; Helsen et al., 1998; Delorme et al., 2011).

### Maturity Effects

In addition to overall age differences, there are substantial hormonally based inter-individual differences in growth and maturation during adolescence (Roemmich and Rogol, 1995; Pearson et al., 2006) and one would expect these differences to influence physical performance (Tanner, 1962; Baxter-Jones, 1995). Maturation also results in an upward movement of the center of mass as the legs lengthen (Aouadi et al., 2012), influencing explosive actions such as sprinting or jumping. Vint and Hinrichs (1996) reported that the maximum height reached during a jump was a product of the height of the center of mass and the position of the body relative to the center of mass at the apex of flight.

However, with the exception of the ability to change direction rapidly (Table 8), multiple regression analyses of the present data set showed no significant contribution of age at peak height velocity, once allowance had been made for calendar age. One factor may have been that many of the players had passed the age of rapid adolescent growth. Morphological characteristics have tended to plateau by the age of 16 to 17 years, at least in European children (Van Praagh and Dore, 2002).

### Influence of Anthropometric Factors

Several authors have discussed the importance of anthropometric variables to the performance of adult handball players (Lidor et al., 2005; Mohamed et al., 2009; Ziv and Lidor, 2009). However, research on adolescent players is limited. Using a stepwise multiple regression analysis, Visnapuu and Jurimae (2007) found that sitting height was associated with scores on basic motor tests fin the 14- to 15-yr.-old group (16.5*–*52.4%; *R*2 × 100) and with specific motor skills in 12- to 13-yr.-olds and 14- to 15-yr.-olds (13.4*–*41.6%; *R*2 × 100). Chamari et al. (2008) previously noted that stride length and sprint performance were proportional to leg length. Aouadi et al. (2012) also reported significant relationships between stature, lower limb length, ratio of lower limb length/stature and sitting height/stature to the jump performance of volleyball players, and Kruger et al. (2014) demonstrated a close relationship between anthropometric data, sprinting, jumping, anaerobic and endurance performance.

Lucia et al. (2002) and Fowkes Godek et al. (2004) underlined the negative effects of excessive fat mass, although the International Handball Federation showed a trend toward the selection of heavier players among the best teams, presumably, the additional mass is here muscle rather than fat particularly in wing players (International Handball Federation, 2014). Handgrip strength is also important for catching and throwing the ball (Nag et al., 2003), and our results showed significant inter-group differences in handgrip performance.

Some studies have demonstrated that body composition influences actual game performance. Handgrip strength gives greater control of the ball, and a higher arm-span allows occupation of greater space in defensive and offensive actions (Fernández et al., 2004). Granados et al. (2007) also demonstrated that a greater fat-free mass was associated with a better performance, because of the increase in the muscular power and strength.

Our univariate data showed substantial correlations between several anthropometric parameters and physical performance, particularly standing height, lower limb length and body mass (Table 5), although the percentage of body fat percentage was not related to any performance measures except vertical and horizontal jumping. Body mass was significantly correlated with the score on all performance tests except vertical and horizontal jumping and 5 and 20 m sprint times. The lack of significant correlation with body mass for these items was surprising. This may possibly reflect differences in familiarity with the CMJA and 5JT. Coordination between the upper and lower limbs is vital to performance of these tests over the age groups studied, with poorer coordination in the younger and less experienced age categories. Further, in multivariate analyses where allowance was made for calendar age, the only statistically significant anthropometric variable was the influence of body mass on ability to change direction (Table 8).

### Playing Position

We did not have a sufficient number of players in any given age category to allow an analysis of our data by playing position. However, technical and physical on-court demands certainly vary with respect to playing positions, and the literature contains data showing such effects in adult players. Wings undertake the greatest amounts of high-intensity running/sprinting, but are involved in fewer one-on-one duels than other players. Pivots cover less distance but are more involved in physical duels and contacts, while backs shoot and pass significantly more compared to the other playing positions (Milanese et al., 2011; Karcher and Buchheit, 2014). These differences lead to differences in anthropometric variables with playing position (Chaouachi et al., 2009; Vila et al., 2012). Chaouachi et al. (2009) demonstrated differences of heights between backs and wings, and in the percentage body fat between goalkeepers and backs in elite Tunisian national handball players. Others have reported that relative to other playing positions wings were significantly lighter and shorter, with less lean body mass and fat mass (Srhoj et al., 2002; Sibila and Pori, 2009; Sporis et al., 2010; Milanese et al., 2011). Sporis et al. (2010) examined a sample of ninety-two elite Croatian handball players, finding that goalkeepers were the oldest, the wings were the shortest and the pivots were the tallest players in the team, while backcourt players had a low percentage of body fat. Ghobadi et al. (2013) also noted that line players (pivots) were the heaviest, backcourt and line players were the tallest, and goalkeepers were older than the center backcourt, backcourt and wing players (*p* < 0.05).

Haugen et al. (2016) quantified differences in both anthropometric and physical characteristics according to playing position and competitive level in elite male handball players. They showed that backs achieved higher throwing velocities than other positions, and wings sprinted faster and jumped higher than pivots and goalkeepers However, back players and wings had greater squat strength than pivots, while pivots were 9% stronger than wing players in 1RM bench press. Massuca et al. (2015) also found significant effects of playing position on body size and fitness performance. Back left/right players had an advantage in handgrip strength, and central back and pivot players also scored better on handgrip strength than goalkeeper and wing players.

### Practical Value of Data on Maturity Status and Anthropometric Characteristics

Our univariate analyses suggest that age, maturity status and anthropometric characteristics all influence scores on performance-related physical tests. However, because a player’s physical characteristics are closely related to age, multiple regression analyses using data that cover the adolescent age range attribute almost all of this variance to age alone. It remains to be demonstrated how far assessments of age, maturity and measurement of anthropometric characteristics can help in player selection, placement and training. In any sport, highly motivated individuals can succeed despite what seems a very unfavorable anthropometric profile, and trainers rely heavily on observing players during actual competition rather than on laboratory data. Nevertheless, these characteristics do seem to influence coaching decisions. Thus, Matthys et al. (2013) noted that youth players with the most advanced maturation status and the most favorable anthropometry and physical fitness scores were consistently positioned in the back position. In contrast, players with a less advanced maturity status and an overall smaller stature were placed on the wing or pivot positions.

### Strengths and Limitations of Study

The main strength of this research is the collection of data on a substantial sample of handball players across age groups that previously have not received great attention. The findings that we report are relevant to the current university population in Tunisia, but we recognize that the rate of attainment of maximal growth differs in other cultures and environments, limiting the generality of our results. Other important limitations include the overwhelming impact of age in the multiple regression analyses, the inability to examine the influence of playing position, and the absence of data on female adolescents. Future observations should focus on a large sample within a single age category, and should include information on performance during actual handball games. Further, we did not assess local muscle mass; this could be a much more interesting variable than total body mass to consider in future investigations. Also, the older and more experienced players had the advantage of having attempted many of the performance tests on previous occasions, and despite familiarization sessions, this may have influenced the scores that they attained relative to the younger players. Other factors that merit consideration in future research include possible effects arising from an age-related displacement of the center of mass, and the development of player position-specific fatigue.

## Conclusion

The present findings underline the progressive age-related development of factors influencing performance throughout adolescence, indicating the importance of age-categorized competition in handball until at least the age of 19 years. The data also showed moderate to very large univariate relationships between the performance realized by both upper and lower limb muscles and the anthropometric characteristics of male handball players, particularly body mass, height and lower limb length. Future studies should focus on narrower age ranges, and should examine the impact of other anthropometric characteristics, such as chest circumference and the length and volume of the upper limbs.

## Ethics Statement

This study was reviewed and approved by the Institute’s Committee on Research for the Medical Sciences (Manouba University Ethics Committee) and performed in accordance with the current national laws and regulations and the Declaration of Helsinki. Informed consent was gained from all participants and their parents or guardians after a verbal and a written explanation of the experimental protocol and its potential risks and benefits. Participants were assured that they could withdraw from the trial without penalty at any time.

## Author Contributions

MC, MH, PC, SH, and RS carried out the formal analysis and supervised the study. HM, NG, and GA investigated the study. HM, NG, and SH developed the methodology. MC and HM administered the project. MH, NG, SH, and MC drafted the manuscript. SH, MH, MC, and RS reviewed and edited the manuscript.

## Conflict of Interest Statement

The authors declare that the research was conducted in the absence of any commercial or financial relationships that could be construed as a potential conflict of interest. The reviewer HW declared a past co-authorship with one of the authors SH to the handling Editor.
